# Preterm birth in twin pregnancies: Clinical outcomes and predictive parameters

**DOI:** 10.12669/pjms.324.10409

**Published:** 2016

**Authors:** Zehra Nihal Dolgun, Cihan Inan, Ahmet Salih Altintas, Sabri Berkem Okten, Niyazi Cenk Sayin

**Affiliations:** 1 Zehra Nihal Dolgun, MD. Department of Obstetrics and Gynecology, Trakya University Medicine Faculty, Edirne, Turkey; 2 Cihan Inan, MD. Department of Obstetrics and Gynecology, Trakya University Medicine Faculty, Edirne, Turkey; 3 Ahmet Salih Altintas, MD. Department of Obstetrics and Gynecology, Trakya University Medicine Faculty, Edirne, Turkey; 4 Sabri Berkem Okten, MD. Department of Obstetrics and Gynecology, Trakya University Medicine Faculty, Edirne, Turkey; 5 Niyazi Cenk Sayin, MD. Department of Obstetrics and Gynecology, Trakya University Medicine Faculty, Edirne, Turkey

**Keywords:** Neonatal, Outcome, Pregnancy, Preterm birth, Twin

## Abstract

**Objective:**

To document the neonatal outcomes of preterm birth in twin pregnancies and to investigate whether perinatal and obstetric parameters are associated with clinical outcomes.

**Methods:**

This retrospective trial was conducted on data gathered from 176 preterm twins delivered in the obstetrics and gynecology department of our tertiary care center. Data extracted from medical files of 88 pregnant women who gave preterm birth (at 26^0/7^ to 36^6/7^ gestational weeks) to twins were analyzed. Maternal/fetal descriptive and obstetric parameters, sonographic data, route of delivery, indication for cesarean section, birth weight, Apgar scores, head circumference, umbilical cord length and placental weight were noted.

**Results:**

The average age of the pregnant women was 28.8±6.4 years and ultrasonographic gestational age was 31.9±2.6 weeks. Apgar scores at 1^st^ minute were affected significantly by fetal body weight (p=0.001), gestational age (p=0.001), height (p=0.004) and head circumference (p=0.011). None of these variables exhibited a noteworthy effect on Apgar scores at 5^th^ minute.

**Conclusion:**

Efforts must be made to achieve advancement of gestational age until delivery in the follow-up preterm of twins. A well-established algorithm with special emphasis to risk factors is necessary to standardize and popularize the appropriate management strategy.

## INTRODUCTION

Popularization of the use of assisted reproductive technologies and the advancement in the average age at first childbirth have contributed to an increased incidence of twin pregnancies worldwide.^1^ Twin deliveries comprise approximately 2-3% of live births.^2^

Twin delivery constitutes a challenge in daily obstetric practice which becomes even more difficult in cases with preterm birth.^3^ Several publications have demonstrated that cesarean delivery may decrease morbidity for term and preterm second twins.^4^-^6^ However, they lack information about maternal-fetal condition on admission and the indication for cesarean or vaginal delivery. Available data were insufficient to allow recommendations to be made about the ideal route of delivery.^1^,^7^ There is debate on the risks of neonatal morbidity and mortality related to complications of vaginal delivery in preterm twin pregnancies. This is particularly a major concern for second twins due to the higher probability of hypoxia after delivery maneuvers, cord prolapse or premature placental separation.^8^,^9^ A recent Cochrane systematic review focusing on the best mode of delivery for preterm infants concluded that recruiting difficulties are likely to make a randomized study on this topic impossible.^10^

In the current study, our purpose was to document the neonatal outcomes of preterm birth in twin pregnancies and to investigate whether perinatal and obstetric parameters are associated with clinical outcomes.

## METHODS

This retrospective study was carried out in the obstetrics & gynecology department of our institution following the approval of local Institutional Review Board.

Data from medical files of 88 pregnant women who gave preterm birth (between 26^0/7^ to 36^6/7^ weeks of gestation) to twins were analyzed. Exclusion criteria were ablatio placenta, placenta previa, twin-to-twin transfusion syndrome, fetal growth restriction (fetal abdominal circumference <10% and estimated fetal weight below 10^th^ percentile), delayed birth of second twin (delivery interval between two twins longer than two hours) and any prenatal diagnosis of malformation of either twin.

Gestational age was determined with respect to the last menstrual date and ultrasonography during the first trimester. In case of discrepancy of more than 5 days between two diagnostic modes, ultrasonographic data was used. Tocolytics were administered up to 33^6/7^ weeks in cases without findings of chorioamnionitis. Antibiotherapy (amoxicillin 2 g/day for a week) was given for premature rupture of membranes and pregnants with preterm labour routinely received corticosteroids to promote fetal pulmonary maturation. All vaginal and ceserean twin deliveries were accompanied by a senior attending obstetrician.

Maternal and gestational ages, gender, route of delivery, indications for cesarean section, birth weight, head circumference, height, placental weight, Apgar scores at 1^st^ and 5^th^ minutes after birth and length of umbilical cord were noted. Associations between Apgar scores and perinatal fetal measurements with descriptive and baseline obstetric features were sought. Apgar score served as a direct measure of perinatal morbidity, while head circumference, birth weight and length of umbilical cord were accepted as indirect indicators.

Analysis of data was made using IBM SPSS Statistics 20 program. Normal distribution of data was tested with Kolmogorov-Smirnov test. Two independent groups were compared by means of Independent-Samples T test and Mann-Whitney U tests. For qualitative variables, frequency and percentage (%) were utilized. Confidence interval was 95% and level of statistical significance was set at p<0.05.

## RESULTS

An overview of descriptive and obstetric characteristics in our series is shown in Table-I. The average age of the pregnant women was 28.8±6.4 (range:14-54) years. Gestational age with respect to the last menstrual date and ultrasonography were 32.8±2.8 weeks (range: 25-36) and 31.9±2.6 weeks (range: 24-36). Cesarean section was performed in 83 deliveries (94.3%), while vaginal delivery occurred in 5 cases (5.7%). In this series of preterm twins, the average birth weight (g), height (cm), head circumference (cm), placental weight (g) and umbilical cord length (cm) were 1979.3±538.0; 45.7±32.7; 30.4±2.6; 590.4±211.5 and 34.5±7.1, respectively.

**Table-I:** Baseline descriptive maternal and perinatal data in our series of preterm twins (maternal age and fetal weight are expressed as mean±standard deviation; gestational age, height, head circumference, placental weight and umbilical cord length are described in median-interquartile range).

Variable		mean±SD (range: minimum-maximum)
Maternal age (years)		28.8±6.4 (14-54)
Gestational age‡(weeks)		32.8-2.8 (25-36)
Gestational age§(weeks)		31.9-2.6 (24-36)
Fetal weight (grams)		1979.3±538.0 (130-3260)
Height (cm)		45.7-32.7 (24-474)
Head circumference (cm)		30.4-2.6 (20-37)
Placental weight (grams)		590.4-211.5 (280-1300)
Umbilical cord length (cm)		34.5-7.1 (20-67)
Route of delivery (n,%)	C/S	83 (94.3)
	Vaginal	5 (5.7)
Gender (n,%)	Male	91 (51.7)
	Female	85 (48.3)
Apgar^1^	7-10 (n,%)	113 (73.4)
	4-6 (n,%)	31 (20.1)
	0-3 (n,%)	10 (6.5)
Apgar^5^	7-10 (n,%)	145 (94.2)
	4-6 (n,%)	1 (0.6)
	0-3 (n,%)	8 (5.2)

***Abbreviations:*** ‡: gestational age according to last menstrual date; §: gestational age according to ultrasonographic measurements; C/S: cesarean section;

Apgar^1^: Apgar score at 1^st^ minute postnatally; Apgar^5^: Apgar score at 5^th^ minute postnatally; SD: standard deviation

Relationship between maternal age, fetal birth weight and Apgar scores on 1^st^ and 5^th^ minutes is shown in Table-II. Analysis of our data demonstrated that Apgar scores at 1^st^ minute were affected significantly by fetal birth weight (p=0.001). Table-III indicates that gestational age (p=0.001), height (p=0.004) and head circumference (p=0.011) seemed to affect Apgar scores at 1^st^ minute significantly. Route of delivery (p=0.612) and gender (p=0.182) did not exhibit a remarkable effect on Apgar scores at 1^st^ minute (Table-IV).

**Table-II:** Impacts of maternal age and fetal weight on Apgar scores at 1^st^ and 5^th^ minutes (For Apgar scores at 5^th^ minute, scores between 0-3 and 4-6 were combined in order to achieve the sufficient number of patients needed for statistical analysis).

Variable	Apgar^1^	mean±SD	p Value	Apgar^5^	mean±SD	p Value
Maternal age (years)	7-10	28.6±6.8	0.843	7-10	28.5±6.7	0.747
	4-6	27.9±6.4		0-6	29.2±3.5	
	0-3	29.1±4.5				
Fetal weight (grams)	7-10	2044.3±484.1	0.001*	7-10	1966.7±511.1	0.173
	4-6	1711.6±497.3		0-6	1718.9±751.8	
	0-3	1658.0±772.6				

***Abbreviations:*** Apgar^1^: Apgar score at 1^st^ minute postnatally 1; Apgar^5^: Apgar score at 5^th^ minute postnatally; *: statistically significant; SD: standard deviation

**Table-III:** Impacts of gestational weeks, height, head circumference, placental weight, length of umbilical cord, gender of the neonate and route of delivery on Apgar scores at 1^st^ minute (variables are expressed as median-interquartile range).

Variable	Apgar^1^			p Value

	7-10	4-6	0-3	
Gestational age ‡ (weeks)	34.0-2.5	32.0-5.7	32.0-4.3	0.032*
Gestational age § (weeks)	32.4-3.0	31.0-5.0	31.0-5.5	0.001*
Height (cm)	44.0-4.0	43.0-5.0	40.0-12.8	0.004*
Head circumference (cm)	31.0-3.0	29.0-4.0	29.5-5.5	0.011*
Placental weight (g)	550.0-270.0	600.0-380.0	520.0-240.0	0.401
Umbilical cord length (cm)	35.0-8.0	32.0-8.0	33.5-10.5	0.333

***Abbreviations:*** ‡: gestational age according to last menstrual date; §: gestational age according to ultrasonographic measurements; Apgar^1^: Apgar score at 1^st^ minute postnatally;

* statistically significant.

**Table-IV:** Impacts of gestational weeks, height, head circumference, placental weight, length of umbilical cord, gender of the neonate and route of delivery on Apgar scores at 5^th^ minute (For Apgar scores at 5^th^ minute, scores between 0-3 and 4-6 were combined in order to achieve the sufficient number of patients needed for statistical analysis; variables are expressed as median-interquartile range).

Variable	Apgar^5^		p Value

	7-10	0-6	
Gestational age‡ (weeks)	34.0-4.0	33.0-4.1	0.233
Gestational age§ (weeks)	32.0-3.4	31.0-6.0	0.102
Height (cm)	44.0-5.0	41.0-11.0	0.220
Head circumference (cm)	31.0-3.0	30.0-4.5	0.975
Placental weight (g)	560.0-270.0	600.0-335.0	0.550
Umbilical cord length (cm)	34.0-8.0	35.0-8.0	0.367

***Abbreviations:*** ‡: gestational age according to last menstrual date; §: gestational age according to ultrasonographic measurements; Apgar^5^: Apgar score at 5^th^ minute postnatally.

Analysis of the impacts of gestational age according to menstrual period (p=0.233) or ultrasonography (p=0.102), height (p=0.220), head circumference (p=0.975), placental weight (p=0.401) and umbilical cord length (p=0.331) did not have a remarkable effect on Apgar scores on 1^st^ minute (Table-IV). Similarly, gender of the newborn (p=0.110) and route of delivery (p=0.562) did not have a noteworthy effect on Apgar scores at 5^th^ minute. Neither weight of placenta (p=0.550), nor length of the umbilical cord (p=0.367) had a noteworthy impact on Apgar scores of twins on 5^th^ minute.

We did not find any significant difference between the firstborn and secondborn twins with respect to Apgar scores on 1^st^ minute (p=0.118) and Apgar scores on 5^th^ minute (p=0.426). Similarly, diagnosis of premature rupture of membranes did not have a remarkable effect on Apgar scores on 1^st^ minute (p=0.135) and on 5^th^ minute (p=0.211).

For the firstborn twins, neonates born ≥32 gestational weeks displayed better Apgar scores on 1^st^ minute (p=0.007) and on 5^th^ minute (p=0.003) compared to firstborn twins delivered after <32 gestational weeks. In the same way, neonates born ≥32 gestational weeks had better Apgar scores on 1^st^ minute (p=0.001) and on 5^th^ minute (p<0.001) than the secondborn twins delivered <32 gestational weeks.

## DISCUSSION

The objective of the present study was to outline the clinical outcomes in preterm births of twin pregnancies and to seek whether any perinatal, surgical and obstetric parameters are related with clinical outcomes. Our results yielded that advancement of gestational age is crucial to achieve acceptable fetal growth rates and better Apgar scores after birth. Particularly, Apgar scores at first minute are more prone to be influenced by perinatal variables.

In 2006, 60% of the twins delivered in the United States were preterm and weighed less than 2500g.^2^ Ultrasonographic examination is important for not only the determination of chorionicity and amnionicity, but also for identification of anomalies and syndromes in twin gestations.^11^ Timely recognition of risk factors and appropriate management measures will decrease the likelihood of adverse pregnancy outcome. Obviously, twin pregnancies are more likely to be delivered preterm than singleton pregnancies.^12^,^13^

Transvaginal cervical length or fetal fibronectin level can be used to distinguish pregnancies that are more likely to deliver prematurely.^14^ It must be remembered that routine use of the diagnostic tests in twin pregnancies would not decrease the actual rate of preterm births.^2^

One of the more common interventions that have been tried in the past was the use of prophylactic oral betamimetics to reduce the incidence of preterm birth in twin gestations.^15^ Tocolysis, progesterone and reinforcement of the cervix with a cerclage has not been proved to have beneficial effects on prenatal birth. Similarly, other prophylactic interventions such as bed rest and home uterine monitoring were not useful for prevention of preterm birth.^2^,^16^,^17^

The use of antenatal corticosteroids has been shown to decrease perinatal mortality, respiratory distress syndrome, necrotizing enterocolitis and systemic infections. However, in spite of the improvement provided by antenatal steroid treatment, it should not be administered repetitively.^18^,^19^

The current retrospective study is noteworthy for several reasons: First, Apgar scores at first minute are affected more remarkably by perinatal factors in preterm twins and this circumstance must be considered during the interpretation of Apgar scores after birth. Determination of gestation age with respect to ultrasonography and menstrual date yielded consistent information and both of these measures can be used for obtaining reliable data. Efforts must be made to allow advancement of pregnancy in preterm twins and achievement of acceptable fetal growth can result in a better prognosis as reflected in Apgar scores at first minute. Our findings highlight that growth restriction and earlier occurence of delivery result in worse Apgar scores. Hence, they are risk factors for neonatal morbidity or mortality. Our results are in agreement to a recent study by Blickstein et al.^20^ They have evaluated the neonatal mortality rate among discordant twins classified according to the birth weight of the smaller twin. The authors showed significantly higher neonatal mortality among twin pairs in whom the smaller twin was small for gestational age. Our results showed that Apgar scores of preterm twins were not influenced by placental weight or length of umbilical cord. These findings imply that other perinatal factors such as infection may have a more critical role for fetal outcome in preterm twin births. We noted that diagnosis of premature rupture of membranes seemed to have no impact on Apgar scores of twins. Therefore, elucidation of factors prone to influence outcomes in preterm births warrants implmentation of further clinical trials on larger series.

Limitations of the study: It is retrospective study and unpreventable impacts of social, economical, personal and ethnic factors on our results. Also the small vaginal birth sample versus high cesarean delivery ratio is another limitation. Furthermore, this data reflects the experience of a single institution and randomization is usually impossible by means of delivery route in such trials due to ethical and legal issues. Therefore, extrapolations and interpretations of our results must be made with caution.

In summary, we suggest that early recognition of risk factors and increased awareness on risks associated with preterm birth in twin pregnancies is important. Efforts must be made to provide advancement of gestational age until delivery to allow the fetal growth as much as possible. A well-established algorithm with special emphasis to these signs is necessary to standardize and popularize the appropriate management strategy.
